# Monte Carlo Wavefunction Approach to Singlet Fission Dynamics of Molecular Aggregates

**DOI:** 10.3390/molecules24030541

**Published:** 2019-02-01

**Authors:** Masayoshi Nakano, Kenji Okada, Takanori Nagami, Takayoshi Tonami, Ryohei Kishi, Yasutaka Kitagawa

**Affiliations:** 1 Department of Materials Engineering Science, Graduate School of Engineering Science, Osaka University, Toyonaka, Osaka 560-8531, Japan; kenji.okada@cheng.es.osaka-u.ac.jp (K.O.); takanori.nagami@cheng.es.osaka-u.ac.jp (T.N.); takayoshi.tonami@cheng.es.osaka-u.ac.jp (T.T.); rkishi@cheng.es.osaka-u.ac.jp (R.K.); kitagawa@cheng.es.osaka-u.ac.jp (Y.K.); 2 Center for Spintronics Research Network (CSRN), Graduate School of Engineering Science, Osaka University, Toyonaka, Osaka 560-8531, Japan; 3 Quantum Information and Quantum Biology Division, Institute for Open and Transdisciplinary Research Initiatives, Osaka University, Toyonaka, Osaka 560-8531, Japan; 4 Institute for Molecular Science, 38 Nishigo-Naka, Myodaiji, Okazaki 444-8585, Japan

**Keywords:** Monte Carlo wavefunction, singlet fission, quantum master equation, molecular aggregate

## Abstract

We have developed a Monte Carlo wavefunction (MCWF) approach to the singlet fission (SF) dynamics of linear aggregate models composed of monomers with weak diradical character. As an example, the SF dynamics for a pentacene dimer model is investigated by considering the intermolecular electronic coupling and the vibronic coupling. By comparing with the results by the quantum master equation (QME) approach, we clarify the dependences of the MCWF results on the time step (Δ*t*) and the number of MC trajectories (*M*_C_). The SF dynamics by the MCWF approach is found to quantitatively (within an error of 0.02% for SF rate and of 0.005% for double-triplet (TT) yield) reproduce that by the QME approach when using a sufficiently small Δ*t* (~0.03 fs) and a sufficiently large *M*_C_ (~10^5^). The computational time (*t*_req_) in the MCWF approach also exhibits dramatic reduction with increasing the size of aggregates (*N*-mers) as compared to that in the QME approach, e.g., ~34 times faster at the 20-mer, and the size-dependence of *t*_req_ shows significant reduction from *N*^5.15^ (QME) to *N*^3.09^ (MCWF). These results demonstrate the promising high performance of the MCWF approach to the SF dynamics in extended multiradical molecular aggregates including a large number of quantum dissipation, e.g., vibronic coupling, modes.

## 1. Introduction

Singlet fission (SF) is a photophysical process, where a singlet exciton state splits into two triplet excitons, and is known to be a very fast reaction process on time scales of pico- or subpico-seconds [1,2,3,4,5]. One of the reasons is that the two triplet excitons created are firstly coupled and in a singlet state overall. The SF materials in solar cells are expected to reduce the energy loss concerning the excess absorption energy by creating another triplet exciton and to increase the number of the created triplet excitons which reach the donor-acceptor interface due to their longer lifetime than singlet excitons [6]. Thus, intensive experimental and theoretical studies on SF have been conducted toward development of efficient single-junction solar cells. Nakano et al. clarified that the molecules suitable for efficient SF materials tend to exhibit weak diradical character (*y*_0_) as well as much weaker tetraradical character (*y*_1_) [7,8,9], i.e., ~0.1 < *y*_0_ < ~0.5 and *y*_1_/*y*_0_ < ~0.2 (at the spin-projected unrestricted Hartree-Fock (PUHF) level of approximation), which are needed to satisfy the energy level matching conditions for the monomer presented by Smith and Michl [1]: (a) $2E(T_{1})\sim E(S_{1})$ or $2E(T_{1})<E(S_{1})$ and (b) $2E(T_{1})<E(T_{2})$, where S_1_ and T_1_ indicate the lowest singlet and triplet exciton states, respectively, and T_2_ indicates the second triplet exciton state. In addition, the investigation of the effects of molecular packing and vibronic coupling on the SF dynamics is also indispensable for understanding the detailed SF mechanism as well as for constructing the rational design guidelines for highly-efficient SF materials [1,2,3,4,5,10,11,12,13,14,15,16,17,18,19]. The SF dynamics is characterized by the SF rate (singlet Frenkel exciton (FE) state (e.g., S_1_S_0_) → double-triplet (correlated triplet-pair) exciton state ^1^(T_1_T_1_), which is referred to as TT hereafter) and TT yield. Although the SF rate is often evaluated by perturbation theory (Fermi’s golden rule) using the electronic couplings calculated for cluster models [17,20], it is known that there are some application limits in such kinetic models [13,20,21]. Thus, non-perturbative approaches such as the time-convolutionless (TCL) quantum master equation (QME) approach [22] are recently applied to the exciton dynamics in oligomers with multiple exciton states [23,24,25]. For SF dynamics in oligomer cases, however, the numerical integration of the QME using the reduced density matrix is known to encounter difficulty in the case of a large number of bases (*N*_B_) since the number of density matrix elements is proportional to $N_{B}^{2}$. One approach to overcome this difficulty is the Monte Carlo wavefunction (MCWF) approach, which has been developed at first in the field of quantum optics by Dalibard, Castin and Mølmer [26] and also by Carmichael [27] for simulating open quantum systems. The dynamics by the MCWF approach is described by both the continuous time-evolution obtained by solving the Schrödinger-type wave equation with non-Hermitian Hamiltonian, and quantum jumps randomly interrupting the coherent evolution of the system. As a result, the MCWF approach generates a large number of quantum trajectories of wavefunctions, and the ensemble average of the quantum trajectories is proved to satisfy the QME under the Markov approximation for the reduced system density operator [26,27]. Due to solving the Schrödinger-type wave equation (not QME), a significant advantage of the MCWF approach lies in its small numerical efforts (the number of elements is proportional to *N*_B_). This advantage is predicted to become marked when we investigate the dynamics in large-scale dissipative quantum systems, e.g., SF dynamics of oligomers with a large number of vibronic coupling modes. 

In this study, the first-order MCWF approach is applied to the SF dynamics of molecular aggregates involving vibronic couplings. We investigate the dependences of the accuracy of the results of SF dynamics (SF rate and TT yield) on time step and the number of MC trajectories using a pentacene dimer model, and discuss the applicability and performance of the MCWF approach to the SF dynamics for larger-size molecular aggregate systems.

## 2. Hamiltonian of a Linear Molecular Aggregate

**Figure 1:**
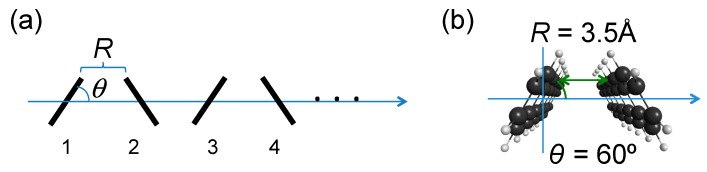
Linear molecular aggregate (*N*-mer) model with monomer number (**a**) and its dimer (*N* = 2) unit (*θ* = 60°, *R* = 3.5 Å) (**b**).

The total Hamiltonian of a linear molecular aggregate (*N*-mer) (Figure 1a) [25]:

(1) $$H=H_{\mathrm{ex}}+H_{\mathrm{ph}}^{}+H_{\mathrm{ex}\text{-}\mathrm{ph}}^{}$$

where *H*_ex_ is the exciton Hamiltonian; *H*_ph_ and *H*_ex-ph_ indicate the phonon (vibrational) Hamiltonian and exciton-phonon (vibronic) coupling Hamiltonian, respectively. In the exciton Hamiltonian *H*_ex_, only the interactions between neighboring monomers are considered, while the direct couplings between FE and TT states, couplings between charge transfer (CT) states and those between TT states are ignored because they are known to be mostly much smaller than the other couplings [1,2]. Thus, the approximate exciton Hamiltonian *H*_ex_ is expressed as [25]

(2) $$\begin{array}{cc} H_{\mathrm{ex}} & =H_{\mathrm{FE}}+H_{\mathrm{CT}}+H_{\mathrm{TT}}+H_{\mathrm{int}}\\ & =\sum_{i=1}^{N}E_{S_{1}S_{0}}^{}F_{i}^{†}F_{i}^{}+\sum_{i=1}^{N-1}\left(V_{}^{S_{1}S_{0}/S_{0}S_{1}}F_{i}^{†}F_{i+1}^{}+h.c.\right)+\sum_{i=1}^{N-1}\left(E_{\mathrm{AC}}^{}C_{i,i+1}^{†}C_{i,i+1}^{}+E_{\mathrm{CA}}^{}C_{i+1,i}^{†}C_{i+1,i}^{}\right)+\sum_{i=1}^{N-1}E_{\mathrm{TT}}^{}T_{i,i+1}^{†}T_{i,i+1}^{}\\ & +\sum_{i=1}^{N-1}\left(V^{S_{1}S_{0}/\mathrm{AC}}F_{i}^{†}C_{i,i+1}+V^{S_{0}S_{1}/\mathrm{CA}}F_{i+1}^{†}C_{i+1,i}+V^{S_{1}S_{0}/\mathrm{CA}}F_{i}^{†}C_{i+1,i}+V^{S_{0}S_{1}/\mathrm{AC}}F_{i+1}^{†}C_{i,i+1}+h.c.\right)\\ & +\sum_{i=1}^{N-1}\left(V^{\mathrm{TT}/\mathrm{AC}}T_{i,i+1}^{†}C_{i,i+1}+V^{\mathrm{TT}/\mathrm{CA}}T_{i,i+1}^{†}C_{i+1,i}+h.c.\right), \end{array}$$

where the first and the second terms in the right-hand side indicate the FE Hamiltonian; the third and the fourth terms represent the CT and TT Hamiltonians, respectively; the remaining terms represent the interactions between different-type exciton states, i.e., FE–CT and TT–CT. Here, $F_{i}^{†}$($F_{i}^{}$) denotes the creation (annihilation) operator for a FE state at the *i*-th monomer; $C_{i,i+1}^{†}$($C_{i,i+1}^{}$) denotes the creation (annihilation) of an anion (A) and a cation (C) at the *i*-th and (*i*+1)-th monomer, respectively; $T_{i,i+1}^{†}$($T_{i,i+1}^{}$) denotes the creation (annihilation) of two triplets over the *i*-th and (*i*+1)-th monomers. The term h.c. stands for the Hermitian conjugate of the terms already included in each parenthesis. In this model, we approximately consider the situation that all correlated triplet pairs are located on adjacent monomers, i.e., the migration of triplet excitons is ignored. For simplicity, since we consider symmetric linear aggregates composed of identical monomers with the same intermonomer distance, FE exciton energies $E_{S_{1}S_{0}}^{}$ at all the monomers are identical with each other (referred to as $E_{\mathrm{FE}}^{}$ hereafter); CT energies $E_{\mathrm{CA}}$ and $E_{\mathrm{AC}}$ at all (*i*, *i*+1)-pair of the monomers are equal to each other (referred to as $E_{\mathrm{CT}}$ hereafter); TT energies $E_{\mathrm{TT}}$ are the same over all the (*i*, *i*+1)-pair of monomers. The $V_{}^{S_{1}S_{0}/S_{0}S_{1}}$ indicates the FE coupling between S_1_S_0_ at the (*i*, *i*+1)-th monomers and S_0_S_1_ at the (*i*, *i*+1)-th monomers, so that $V_{}^{S_{1}S_{0}/S_{0}S_{1}}=V_{}^{S_{0}S_{1}/S_{1}S_{0}}(\equiv V_{\mathrm{ex}}^{})$ in the present case. For the FE–CT couplings, there are two types of couplings, $V^{S_{1}S_{0}/\mathrm{AC}}=V^{S_{0}S_{1}/\mathrm{CA}}$ and $V^{S_{1}S_{0}/\mathrm{CA}}=V^{S_{0}S_{1}/\mathrm{AC}}$. Similarly, $V^{\mathrm{TT}/\mathrm{CA}}=V^{\mathrm{TT}/\mathrm{AC}}$ is considered for the TT–CT couplings. 

The vibrational and vibronic coupling Hamiltonians are represented in atomic units (*m* = *c* = ℏ = 1 a.u.), respectively, by [24,25]:

(3) $$H_{\mathrm{ph}}^{}=\sum_{a}\omega_{a}^{}b_{a}^{†}b_{a}^{},\;\mathrm{and}\;H_{\mathrm{ex}\text{-}\mathrm{ph}}^{}=\sum_{m}\sum_{a}|m\rangle \langle m|\omega_{a}^{}\left(g_{ma}^{}b_{a}^{}+g_{ma}^{\;*}b_{a}^{\;†}\right)$$

Here, the vibrational Hamiltonian *H*_ph_ is described by a collection of harmonic oscillations, and $b_{a}^{†}$ ($b_{a}^{}$) indicates the creation (annihilation) operator of the *a*-th vibrational mode with a frequency $\omega_{a}^{}$, where the vibrational modes are approximated to be common for each diabatic exciton state. In the vibronic Hamiltonian *H*_ex-ph_, the sum of *m* covers all the diabatic exciton states and *a* runs over all the vibrational modes, where *g_ma_* indicates the coupling constant between diabatic exciton state *m* (with energy ω*_m_*) and vibrational mode *a* (with energy ω*_a_*). In this study, we consider only the Holstein coupling, which causes the fluctuation of the energy gaps among the FE, CT and TT states and is predicted to provide significant effects on the SF dynamics [14,24,25]. Note here that the MCWF can be applied to another type of vibronic coupling, i.e., the Peierls coupling [12], which causes a fluctuation of electronic coupling (off-diagonal term in the exciton Hamiltonian *H*_ex_ matrix) and is mostly a function of intermolecular vibrational modes. The diabatic exciton bases for FE, CT and TT states of a linear *N*-mer model are defined as:

(4) $$\left\{|\mathrm{FE}\rangle \}=\{|S_{1}S_{0}\cdots \rangle ,\;|S_{0}S_{1}\cdots \rangle ,\;\cdots ,\;|\cdots S_{0}S_{1}S_{0}\rangle ,\;|\cdots S_{0}S_{0}S_{1}\rangle \right\}$$

(5) $$\left\{|\mathrm{CT}\rangle \}=\{|{\mathrm{CAS}}_{0}\cdots \rangle ,\;|{\mathrm{ACS}}_{0}\cdots \rangle ,\;|S_{0}{\mathrm{CAS}}_{0}\cdots \rangle ,\;|S_{0}{\mathrm{ACS}}_{0}\cdots \rangle ,\;\cdots ,\;|\cdots S_{0}\mathrm{CA}\rangle ,\;|\cdots S_{0}\mathrm{AC}\rangle \right\}$$

(6) $$\left\{|\mathrm{TT}\rangle \}=\{|{\mathrm{TTS}}_{0}\cdots \rangle ,\;|S_{0}{\mathrm{TTS}}_{0}\cdots \rangle ,\;\cdots ,\;|\cdots \mathrm{TT}\rangle \right\}$$

The numbers of FE, CT and TT bases for the linear *N*-mer model are *N*, 2(*N* – 1), and *N* – 1, respectively (total basis number *N*_B_ = 4*N* − 3). The electronic couplings between those diabatic bases are obtained from those for a dimer system in this study (see Figure 1b) [25]. 

## 3. Quantum Master Equation Approach and Monte Carlo Wavefunction Approach

The second-order time-convolutionless (TCL) QME under the Markov approximation is expressed by [22,23,24,25]:

(7) $$\begin{array}{cc} \frac{d}{dt}\rho (t)= & -i\left[H_{S},\;\rho (t)\right]\\ & -\frac{1}{2}\sum_{m,\omega }\gamma_{m}^{}(\omega )\left(A_{m}^{†}(\omega )A_{m}^{}(\omega )\rho (t)+\rho (t)A_{m}^{†}(\omega )A_{m}^{}(\omega )\right)+\sum_{m,\omega }\gamma_{m}^{}(\omega )A_{m}^{}(\omega )\rho (t)A_{m}^{†}(\omega ), \end{array}$$

where *m* indicates the diabatic exciton state (Equations (4)–(6)); $A_{m}^{}(\omega )=\sum_{E_{q}-E_{p}=\omega }|p\rangle \langle p|A_{m}^{}|q\rangle \langle q|$, where $A_{m}\equiv |m\rangle \langle m|$, and {$|p\rangle (=\sum_{m}C_{mp}|m\rangle )$, $E_{p}$} indicates an eigenstate (adiabatic exciton state) and an eigenvalue of $H_{\mathrm{ex}}|p\rangle =E_{p}|p\rangle$. The second and the third terms on the right-hand side of Equation (7) indicate the relaxation of exciton density matrix (causing SF), which is characterized by the relaxation parameter $\gamma_{m}^{}(\omega )$ under the Markov approximation [25]:

(8) $$\begin{array}{cc} \gamma_{m}^{}(\omega )=2\pi J_{m}^{}(\omega )\left(1+n_{B}(\omega ,T)\right) & \;\mathrm{for}\;\omega \;>\;0,\\ \gamma_{m}^{}(\omega )=2\pi J_{m}^{}(-\omega )n_{B}(-\omega ,T) & \;\mathrm{for}\;\omega \;<\;0,\\ \gamma_{m}^{}(\omega )=4\frac{\lambda_{m}^{}}{\omega_{m\;c}^{}}k_{B}T & \;\mathrm{for}\;\omega \;=\;0, \end{array}$$

where $n_{B}(\omega ,T)$ is the Bose-Einstein distribution of phonons with energy ω at temperature *T*, *k*_B_ is the Boltzmann constant, and $J_{m}^{}(\omega )$ indicates the spectral density of the Holstein vibrational mode of the *m*-th diabatic exciton state. We employ an Ohmic function with a Lorentz-Drude cutoff [14,22,23,24,25]:

(9) $$J_{m}^{}(\omega )=\frac{1}{\pi }\frac{2\lambda_{m}^{}\Omega_{m}^{}\omega }{\omega^{2}+{\left(\Omega_{m}^{}\right)}^{2}}$$

where *λ_m_* and *Ω_m_* indicate the reorganization energy and the cutoff frequency, respectively, in the *m*-th diabatic exciton state. Note here that this spectral density indicates a vibronic coupling distribution with a peak value of *λ_m_*/π at *Ω_m_* vibrational mode. In this study, we consider an identical spectral density case ($\lambda \equiv \lambda_{m}$, $\Omega \equiv \Omega_{m}$) for different diabatic exciton states since our purpose is to just compare the results between the QME and MCWF approaches though the effects of state-dependent spectral densities are discussed in our previous paper [24]. Using Equation (7), the working equations to solve for diagonal and off-diagonal (*p* < *q*) density matrix elements in the representation of the adiabatic exciton basis {|p〉} are given by:

(10) $$\frac{d}{dt}\rho_{pp}(t)=-\sum_{r}\Gamma_{pp;rr}^{}\rho_{rr}(t)$$

(11) $$\frac{d}{dt}\rho_{pq}(t)=-i\omega_{pq}\rho_{pq}(t)-\sum_{r,s}\Gamma_{pq;rs}^{}\rho_{rs}(t)$$

where $\omega_{pq}\equiv \omega_{p}-\omega_{q}$ and decay rate $\Gamma_{pq;rs}^{}$ is expressed as

(12) $$\begin{array}{cc} \Gamma_{pq;rs}^{} & =\frac{1}{2}\sum_{t}\sum_{m}\delta_{sq}{\left|C_{mt}^{}\right|}^{2}C_{mp}^{*}C_{mr}^{}\gamma_{m}^{}(\omega )\delta_{\omega_{pt},\omega }\delta_{\omega_{rt},\omega }+\frac{1}{2}\sum_{t}\sum_{m}\delta_{rp}{\left|C_{mt}^{}\right|}^{2}C_{ms}^{*}C_{mq}^{}\gamma_{m}^{}(\omega )\delta_{\omega_{st},\omega }\delta_{\omega_{qt},\omega }\\ & -\sum_{m}C_{mp}^{*}C_{mr}^{}C_{mq}^{}C_{ms}^{*}\gamma_{m}^{}(\omega )\delta_{\omega_{rp},\omega }\delta_{\omega_{sq},\omega }. \end{array}$$

As seen from these equations, the computational complexity (numerical efforts) of the QME approach is approximately proportional to *N*^5^ (*N*_B_ (the number of bases) is the same order as the number of monomers (*N*) in the present case) since the number of density matrix elements is proportional to *N*^2^ and threefold iteration loops (∝*N*^3^) are included in the right-hand side of Equation (11). This fact is used later in the comparison of computational times for SF dynamics in aggregates between the QME and MCWF approaches. 

In the first-order MCWF approach, the explicit form of Lindblad operator $L_{\mathrm{relax}}$ [26,27] is needed. This describes the relaxation of reduced exciton density (the second and the third terms in Equation (7)), and is expressed under the Markov approximation by [22,23,24,25,26,27]:

(13) $$L_{\mathrm{relax}}\rho (t)\equiv -\frac{1}{2}\sum_{i}\left(C_{i}^{†}C_{i}\rho (t)+\rho (t)C_{i}^{†}C_{i}\right)+\sum_{i}C_{i}^{}\rho (t)C_{i}^{†}$$

Note here that in principle, the MCWF approach can be applied to the QME with the Lindblad-type relaxation term Equation (13) in the Markov approximation. From the integration of the QME (Equation (7)) to the first order in *δt*, the following form is obtained [26,27]:

(14) $$\rho (t+\delta t)≅U\rho (t)U^{†}+\delta t\sum_{i}C_{i}\rho (t)C_{i}^{†}+O(\delta t^{2})$$

where *U* indicates the non-Hermitian evolution (referred to as the “no-quantum-jump” evolution) under the influence of the effective Hamiltonian *H*_eff_:

(15) $$U=\exp\left(-iH_{\mathrm{eff}}\delta t\right),\;\mathrm{where}\;H_{\mathrm{eff}}=H_{S}-\frac{i}{2}\sum_{i}C_{i}^{†}C_{i}$$

Each term on the right-hand side of Equation (14) represents the “minitrajectories” [26,27]. The MCWF approach simulates the evolution of quantum trajectories in Hilbert space conditioned on continuous photodetection involving two types of elements: one is smooth evolution (“no-quantum-jump” evolution) by the non-Hermitian Hamiltonian *H*_eff_, which originates in the first two terms on the right-hand side of Equation (7), and another represents the random interruptions of the non-Hermitian evolution by projections (quantum jumps) described by the second term on the right-hand side of Equation (14) (or the third term in Equation (7)). These two types of evolutions are described by:

(16) |Ψ(t)〉→U|Ψ(t)〉,  (no-quantum-jump)

(17) $$|\Psi (t)\rangle \to C_{i}|\Psi (t)\rangle .\;\;(i\;=\;1,\;2,\;\ldots )\;\;(\mathrm{quantum}\;\mathrm{jump})$$

Note here that the MCWF approach can only treat wavefunctions instead of density matrices in order to obtain the solutions of the QME (Equation (7)). This implies that the MCWF approach requires less computational resources than a numerical integration of the QME, though alternative calculations of a large number of quantum trajectories are needed before an average in the Monte Carlo approach to obtain sufficiently converged solutions of Equation (7). However, since the generation of quantum trajectories is completely independent of each other, the use of parallel computation can overcome this difficulty. As a result, the MCWF approach is expected to be a highly-efficient simulation scheme for treating large-scale open quantum systems involving a large number of degrees of freedom of the system and reservoirs, e.g., exciton states and vibrational modes. 

In the MCWF approach, the density matrix evolution can be simulated with pure states such as Equations (16) and (17) by using an expansion of density matrix into minitrajectories (see Equation (14)). The first minitrajectory (the first term) of Equation (14) (m1) describes a no-quantum-jump evolution and the second (m2) minitrajectories represent quantum jumps. It is noted that the first-order unraveling specifies only one point in the interval δ*t* to condition the density operator by quantum jumps. The procedure of turning Equation (14) into a Monte Carlo simulation is obvious because each minitrajectory in Equation (14) corresponds to the conditioned evolution of the system, which occurs with a specific probability. Thus, the first-order MCWF procedure is described as follows [26,27]:

(i) A random number uniformly distributed between 0 and 1 is generated to choose a minitrajectory (representing no-quantum-jump and/or quantum-jump evolutions of the system) with a specific probability δ*p*_1_ at the next time step δ*t*. 

(ii) The no-quantum-jump evolution is tested first because the probabilities of choosing other minitrajectories (m2*i*) (involving quantum-jumps) are small for small δ*t*. If the no-quantum-jump minitrajectory (m1) is not chosen, one of the minitrajectories (m2*i*) involving quantum-jumps is chosen at the specific probability δ*p*_2*i*_. After the evolution δ*t* of wavefunction for a chosen minitrajectory, the resulting wavefunction is renormalized.

For minitrajectory m1:

(18) $$\mathrm{Wavefunction}\;|wf(1)\rangle =\frac{U|\psi (t)\rangle }{\sqrt{\delta p_{1}}}$$

(19) $$\mathrm{Probability}\;\delta p_{1}=\langle \psi (t)|U^{†}U|\psi (t)\rangle$$

For minitrajectory m2*i* corresponding to *C_i_* (Equation (17)):

(20) $$\mathrm{Wavefunction}\;|wf(2i)\rangle =\frac{C_{i}|\psi (t)\rangle }{\sqrt{\delta p_{2i}/\delta t}}$$

(21) $$\mathrm{Probability}\;\delta p_{2i}=\langle \psi (t)|C_{i}^{†}C_{i}^{}|\psi (t)\rangle \delta t$$

(iii) The procedure (i)–(ii) is repeated at each time step δ*t*.

From Equations (7) and (13), the explicit forms of Lindblad operators *C_i_* in Equation (13) are given by:

(22) $$C_{i}=\sqrt{\gamma_{m}^{}(\omega )}A_{m}^{}(\omega )$$

where *i* represents (*m*, ω). 

## 4. Comparison of QME and MCWF Approaches to SF Dynamics in a Pentacene Dimer Model

In order to clarify the performance of the MCWF approach by comparing with the QME results, we examine a pentacene dimer model with *R* = 3.5 Å and *θ* = 60° (*N* = 2 in Figure 1) [24,25], which indicate the intermonomer distance between the nearest neighbor carbon atoms in the zigzag edges, and the angle between the pentacene monomer plane and the longitudinal axis in parallel to the *R* direction. The monomer geometry is optimized by the RB3LYP/cc-pVDZ method [25] and is employed in the dimer model since the present study is just focused on the comparison between the MCWF and QME results. The *H*_ex_ for the dimer model is expressed in the representation of diabatic exciton basis by:

(23) $$\begin{array}{ll} & \;\;\;|S_{1}S_{0}\rangle \;|S_{0}S_{1}\rangle \;\;\;\;|\mathrm{CA}\rangle \;\;\;\;\;\;\;\;|\mathrm{AC}\rangle \;\;\;\;\;\;\;\;\;|\mathrm{TT}\rangle \;\\ H_{\mathrm{ex}}= & \left(\begin{array}{ccccc} E_{\mathrm{FE}} & V_{\mathrm{ex}} & V_{ll} & -V_{hh} & 0\\ V_{\mathrm{ex}} & E_{\mathrm{FE}} & -V_{hh} & V_{ll} & 0\\ V_{ll} & -V_{hh} & E_{\mathrm{CT}} & 0 & \sqrt{3/2}V_{lh}\\ -V_{hh} & V_{ll} & 0 & E_{\mathrm{CT}} & \sqrt{3/2}V_{hl}\\ 0 & 0 & \sqrt{3/2}V_{lh} & \sqrt{3/2}V_{hl} & E_{\mathrm{TT}} \end{array}\right) \end{array}$$

The electronic couplings are calculated by the following equations [1,2]:

(24) $$V^{S_{1}S_{0}/\mathrm{CA}}\equiv \langle \mathrm{CA}|H_{\mathrm{ex}}|S_{1}S_{0}\rangle \approx \langle l_{A}|F|l_{B}\rangle =V_{ll}$$

(25) $$V^{S_{1}S_{0}/\mathrm{AC}}\equiv \langle \mathrm{AC}|H_{\mathrm{ex}}|S_{1}S_{0}\rangle \approx -\langle h_{A}|F|h_{B}\rangle =-V_{hh}$$

(26) $$V^{\mathrm{TT}/\mathrm{CA}}\equiv \langle \mathrm{TT}|H_{\mathrm{ex}}|\mathrm{CA}\rangle \approx \sqrt{\frac{3}{2}}\langle l_{A}|F|h_{B}\rangle =\sqrt{\frac{3}{2}}V_{lh}$$

(27) $$V^{\mathrm{TT}/\mathrm{AC}}\equiv \langle \mathrm{TT}|H_{\mathrm{ex}}|\mathrm{AC}\rangle \approx \sqrt{\frac{3}{2}}\langle h_{A}|F|l_{B}\rangle =\sqrt{\frac{3}{2}}V_{hl}$$

The FE coupling *V*_ex_ is calculated using the transition densities of the monomers in the Mulliken approximation [25]:

(28) $$V_{\mathrm{ex}}=\langle S_{1}^{}S_{0}^{}|H_{\mathrm{ex}}|S_{0}^{}S_{1}^{}\rangle \approx \sum_{m\in A}\sum_{n\in B}\frac{\rho_{m}^{}\rho_{n}^{}}{r_{mn}}$$

where $\rho_{m}^{}$ and $\rho_{n}^{}$ are the integrated transition densities at atom sites *m* (in monomer A ($h_{A}\to l_{A}$)) and *n* (in monomer B ($h_{B}\to l_{B}$)), respectively, at the B3LYP/cc-pVDZ level of approximation and *r_mn_* is the distance between *m* (in monomer A) and *n* (in monomer B) sites. Here, *h_X_* and *l_Y_* indicate the HOMO (= the highest occupied molecular orbital) and LUMO (= the lowest unoccupied molecular orbital) of monomer *X* and *Y*, respectively, and we assume mutually orthogonal frontier MOs in Equations (24)–(27), so that they can be represented by the Fock matrix $\langle i|F|j\rangle (\equiv V_{ij})$ at the B3LYP/cc-pVDZ level of approximation [1,2,24,25]. For *E*_FE_ and *E*_TT_, we adopt typical values (*E*_FE_ = 2120 meV, *E*_TT_ = 1720 meV) estimated from experiments for pentacene monomer, dimer and crystal [28,29,30]. The CT exciton energy *E*_CT_ for the dimer model is approximately calculated by [24,25]:

(29) $$E_{\mathrm{CT}}\approx E_{C}+E_{A}-2E_{N}+E_{\mathrm{static}}$$

where *E*_C_, *E*_A_, and *E*_N_ represent the self-consistent-field (SCF) energies of the C, A and neutral (N) states, respectively, of the monomer at the B3LYP/cc-pVDZ level of approximation. The electrostatic interaction between the C and A monomers in the dimer configuration is evaluated by [24,25]:

(30) $$E_{\mathrm{static}}\approx \sum_{m\in C}^{}\sum_{n\in A}^{}\frac{q_{m}q_{n}}{r_{mn}}$$

where *q_m_* and *q_n_* indicate the Mulliken atomic charges at atom sites *m* (in C monomer) and *n* (in A monomer), respectively, and *r_mn_* is the distance between sites *m* and *n*. These quantum chemical calculations were performed by Gaussian 09 [31]. The pentacene monomer is shown to give intermediate diradical character *y*_0_ = 0.415 as well as much smaller tetraradical character *y*_1_ = 0.064 at the PUHF/6-31G* level of approximation, and the energy level matching condition is found to be satisfied (*E*_FE_ (2120 meV) > *E*_TT_ (1720 meV)) [25]. Although this diradical character *y*_0_ of a pentacene monomer may be considered to be a little bit larger than expected by experimentalists, it should be noted that the diradical character is a non-observable chemical index and somewhat depends on the calculation method [32,33]. The important point is that the PUHF diradical character map is found to be useful for quantitative screening of efficient SF molecules [7,8]. Indeed, the present results are in good agreement with our diradical-character-based guideline for efficient SF molecules as mentioned in Introduction. The CT exciton state energy *E*_CT_ (2806 meV) calculated using Equation (29) is found to be much higher than the *E*_FE_ and *E*_TT_ in the present dimer model. This indicates that the SF process is driven by the CT-mediated superexchange mechanism as shown in realistic pentacene crystals [1,2]. We employ the electronic couplings (*V_hh_*, *V_ll_*, *V_hl_*, *V_lh_*) = (312.2, −244.7, −247.6, 247.6) meV (Equations (24)–(27)) [25], and the FE coupling *V*_ex_, −34.22 meV (Equation (28)) [25], which is much smaller in amplitude than the other electronic couplings. This indicates that the FE coupling effect on the SF dynamics in the present dimer is not significant, which is in qualitative agreement with our previous results on a realistic pentacene dimer model [24]. The features of relative amplitudes of these electronic couplings, the relative adiabatic exciton energies and the involved diabatic configurations for the dimer model are explained by the different representation of *H*_ex_ using the superposition exciton basis, e.g., superposition FE states = $\left(|S_{1}S_{0}\rangle \pm |S_{0}S_{1}\rangle \right)/\sqrt{2}$ [1,2,24,25]. 

**Figure 2:**
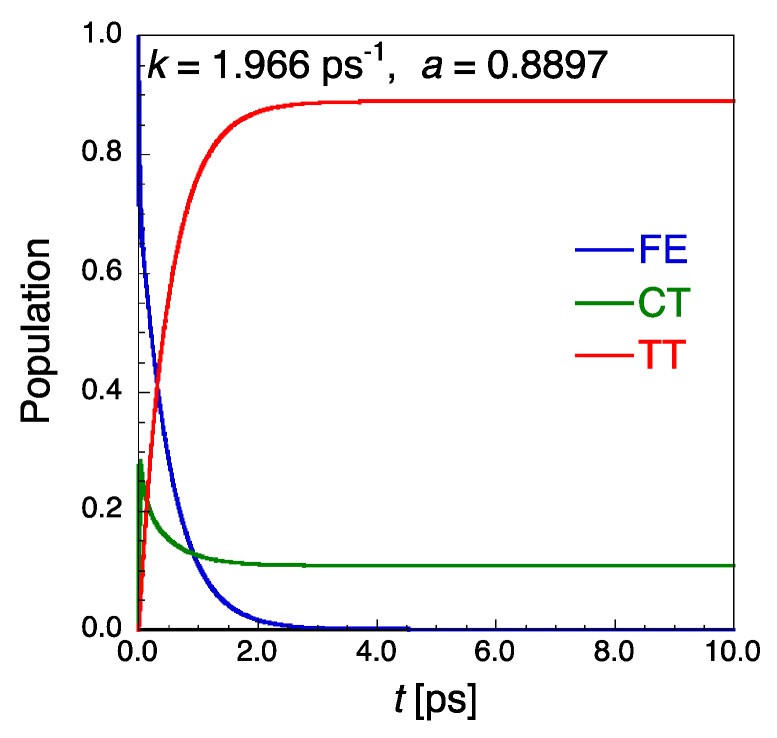
Time-evolution of diabatic exciton state {FE, CT, TT} populations for the pentacene dimer model (Figure 1b) with FE coupling *V*_ex_ = −34.22 meV by the QME approach. The SF rate *k* [ps^−1^] and TT yield *a* [−] are also shown.

Firstly, we show the results of SF dynamics by the QME approach using the six-order Runge-Kutta method. The time step used in the Runge-Kutta method is determined by *Δt* = *T*/*N*_D_, where *T* is a period of a virtual oscillating optical field with a frequency ω = 200 meV, i.e., 20.68 fs, and *N*_D_ is a division number of the field period. Figure 2 shows the time-evolution of diabatic exciton state {FE, CT, TT} populations for the pentacene dimer model with the FE coupling *V*_ex_ = −34.22 meV. The initial population is set to be localized in monomer 1 (Figure 1), i.e., *P*_S1S0_ = 1.0, at *T* = 300 K. The vibronic coupling parameters in the spectral density Equation (9) are set to (λ, Ω) = (50, 180) meV, which are known to be typical values concerning the carbon-carbon stretching mode for acenes and other conjugated organic molecules [14]. The TT yield *P*_TT_ = 1.0 at *t* = ∞ ps means that a singlet exciton created at the initial time (*P*_FE_ = 1.0 at *t* = 0) is converted completely to the double-triplet exciton. The SF rate *k* [ps^−1^] and TT yield (*a*) of SF dynamics are calculated by fitting the time-dependent *P*_TT_ with a three-parameter function $P_{\mathrm{TT}}(t)=a-b\exp(-kt)$ within the first 10 ps, where note that since *b* = *a* − *P*_TT_(0), *b* = *a* is satisfied within the numerical fitting error since the initial population of S_1_S_0_ is 1.0 in the present case. The present dimer model is found to provide *k* = 1.966 ps^−1^ and *a* = 0.8897, the former of which is of the same order as the experimentally observed SF time scales in pentacene [34,35]. It is here noted that the use of *N*_D_ = 40 presents sufficiently precise *k* (converged to the third digit after the decimal point) and TT yield (converged to the fourth digit after the decimal point) in the QME approach to the present system.

**Figure 3:**
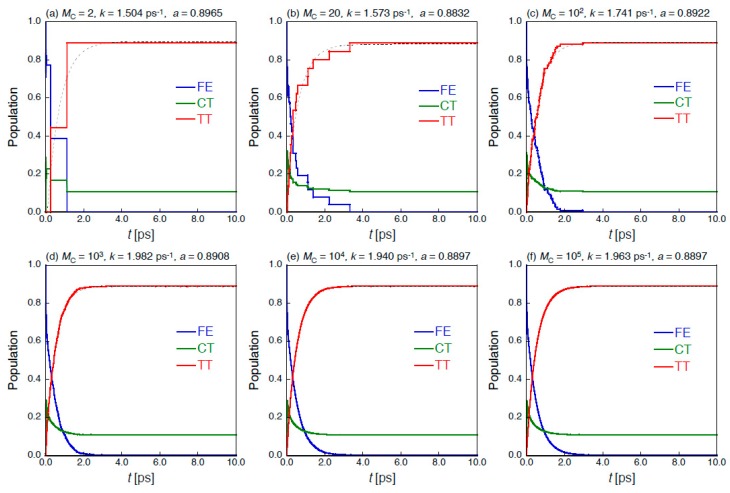
Ensemble results of MCWF time-evolution of diabatic exciton state {FE, CT, TT} populations for the pentacene dimer model (Figure 1b) with FE coupling *V*_ex_ = −34.22 meV with respect to different Monte Carlo sample sizes (*M*_C_). The time step is Δ*t* = 20.68/700 fs ~ 0.03 fs. The estimated SF rate *k* [ps^−1^] and TT yield *a* [−] are also shown with the dotted fitting curves (*P*(TT) = *a* – *b*exp(−*kt*)).

On the other hand, the numerical error of the result obtained by the MCWF approach is known to depend on the time step used in the Runge-Kutta method to perform the no-quantum-jump evolution Equation (16), and the sample size *M*_C_ of the Monte Carlo trajectories used in the Monte Carlo ensemble. The former effect of the time step is alternatively examined by the division number *N*_D_, i.e., *Δt* = 20.68/*N*_D_ fs. Figure 3 shows the convergence behaviors of each diabatic exciton population for the dimer with respect to the different *M*_C_ at *N*_D_ = 700. It is turned out that when the *M*_C_ is not large enough (Figure 3a–c), the time evolution of exciton population changes stepwise. This indicates that the stochastic interruptions (quantum jumps) of continuous time evolution occur in the MCWF scheme, and that we need more Monte Carlo trajectories to obtain the sufficiently converged results. When *M*_C_ is larger than ~10^4^ (Figure 3e,f), the dynamical behavior of exciton population is in good agreement with that obtained by the QME approach (see Figure 2).

**Figure 4:**
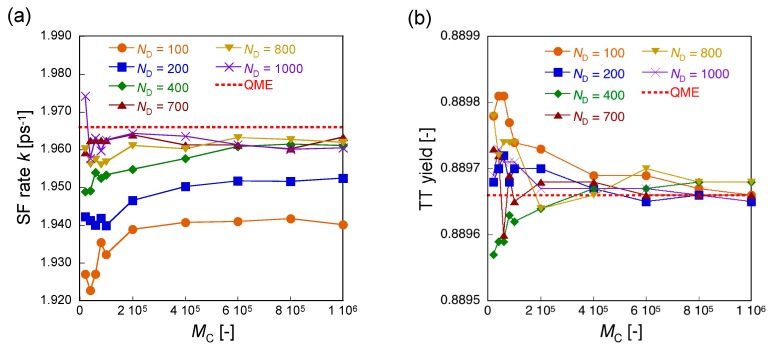
Variations of SF rate *k* [ps^−1^] (**a**) and TT yield *a* [−] (**b**) for the pentacene dimer model (Figure 1b) as a function of the number of Monte Carlo trajectories (*M*_C_) for different division number *N*_D_. For (**a**) and (**b**), the horizontal dotted lines show *k* = 1.966 ps^−1^ and *a* = 0.88966, respectively, obtained by the QME approach.

We here examine the quantitative dependences of the calculated SF rate *k* and TT yield for the present pentacene dimer on the number of *M*_C_ for different division numbers (*N*_D_), where the time step is Δ*t* = 20.68/*N*_D_ fs (see Figure 4). It is found that the error of SF rate tends to reduce with increasing the *M*_C_ for *N*_D_ = 100–400 though the converged SF rates are improved as increasing *N*_D_: the SF rates achieve the converged values approaching to 99% (*N*_D_ = 100), 99.5% (*N*_D_ = 200), and 99.8% (*N*_D_ = 400) of 1.966 ps^−1^ (QME) around *M*_C_ = 6 × 10^5^. It is found for *N*_D_ ≥ 700 that the error of SF rate is rapidly reduced before *M*_C_ = 2 × 10^5^, and after *M*_C_ = 5 × 10^5^, it remains around 99.8% of 1.966 ps^−1^ (QME). The converged values of TT yields are found to show much smaller errors (<0.0045%) than those of the SF rates after *M*_C_ = 4 × 10^5^. 

**Figure 5:**
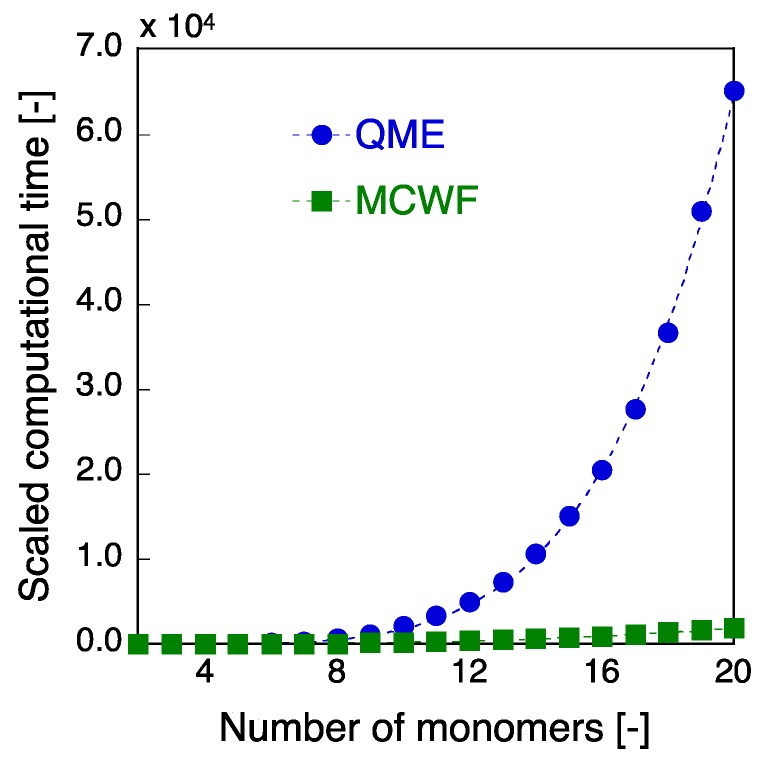
Computational time (*t*_req_) vs. the size (*N*: the number of monomers) of the linear pentacene aggregate model by the QME (with *N*_D_ = 40) and MCWF (with *N*_D_ = 700) approaches. All the times are scaled with the computational time at *N* = 2 (MCWF) as the reference value of 1.0. The fitting curves *t*_req_ = α*N*^β^ are also shown by dotted curves ((α,β) ~ (0.013, 5.15) for the QME vs. (0.188, 3.09) for the MCWF).

We finally discuss the performance of the MCWF approach as compared to the conventional QME approach to the SF dynamics. As easily predicted from the difference in the calculation schemes, the numerical effort in the MCWF approach is expected to be significantly reduced as compared to the QME approach since the MCWF treats a wavefunction instead of a density matrix. In the case of a large number of *M*_C_, the ensemble average of the trajectories can be performed without difficulty using the distributed processing since the calculation of each trajectory is definitely independent of each other. In addition, although the one-time step evolution generally involves the evaluation of plural minitrajectories (*N*_min_: the number of minitrajectories) [see Equations (18) and (20)], the calculation of all the minitrajectories is usually unnecessary since the probability of no-quantum-jump, i.e., non-Hermitian continuous evolution, is larger than those of quantum jumps in the case of usual vibronic couplings. Even if not so, these minitrajectories are also independent of each other, so that the calculations of probabilities of the minitrajectories can be partitioned into each node computer, providing all the probabilities in one-time step at a time. Thus, such distributed computing (by partitioning into *M*_C_ × *N*_min_ nodes of computers, in principle) enables to perform the MCWF approach with a significantly reduced computational power as compared to the conventional QME approach. The computational times required for the SF dynamics (up to 500 optical cycle (~10 ps)) in the present linear pentacene *N*-mer model (*N* = 2–20) are shown in Figure 5, where the time (*t*_req_) required for a Monte Carlo trajectory (*M*_C_ = 1) is considered for the MCWF approach, and all the required times are scaled with that at *N* = 2 (MCWF) as the reference value of 1.0. Here, we adopt the time steps Δ*t* = 20.68/40 ~ 0.517 fs for the QME and 20.68/700 ~ 0.03 fs for the MCWF, both of which are found to give sufficiently converged SF rate and TT yield with the similar precision (see Figure 2 and Figure 4). Although for small size systems (*N* ≤ 3), the required time is found to be larger in the MCWF than in the QME, e.g., *t*_req_ = 0.0399 (MCWF) vs. 0.0236 (QME) at *N* = 2, the required time in the QME is shown to remarkably increase with increasing *N* (*N* > 3) as compared to that in the MCWF, e.g., ~34 times speed up at *N* = 20 by the MCWF approach. Indeed, the size (*N*) dependence of *t*_req_ for the QME is found to be much larger than that for the MCWF: *t*_req_ ∝ *N*^5.15^ (QME) vs. *t*_req_ ∝ *N*^3.09^ (MCWF). The size dependence of *t*_req_ in the QME approach is found to be in good agreement with the computational complexity estimated in Equations (10)–(12) though the exponent ratio QME/MCWF = 5.15/3.09 ~ 1.66 is slightly smaller than that (2.0) expected from the relationship of the number of elements between these two approaches, *N*_QME_ = (*N*_MCWF_)^2^. This is predicted to be caused by the increase in the trial numbers of minitrajectories (*N*_min_) generated by quantum jumps in each time step in the MCWF approach (Equation (20)) since the present MCWF calculations are done by only partitioning into *M*c nodes of computers. Thus, the performance of the MCWF approach is expected to be further improved in principle by partitioning into *M*_C_ × *N*_min_ nodes of computers. In summary, the present results demonstrate the outstanding advantage of the MCWF approach over the conventional QME approach when applying to the SF dynamics of extended molecular aggregate systems with a large number of vibronic coupling modes by partitioning the calculations of trajectories into the *M*_C_ (× *N*_min_) nodes of computers in the MCWF approach.

## 5. Concluding Remarks

We have developed the MCWF approach to the SF dynamics of linear molecular aggregate systems involving the Holstein vibronic couplings approximated by an Ohmic function with a Lorentz-Drude cutoff. The SF dynamics obtained by the MCWF approach is found to reproduce the QME results when we employ a high-order Runge-Kutta method with a sufficiently small time step for the continuous non-Hermitian time-evolution and a sufficiently large number of Monte Carlo trajectories for the ensemble average. It is found that the increase in the numerical efforts with the increase in the size of the system is significantly reduced by distributing the calculations of Monte Carlo trajectories to a sufficient number of nodes of computers since the calculation of trajectories is independent of each other. In summary, the MCWF approach is expected to be indispensable for the analysis of the SF mechanism and rational design of highly-efficient SF materials since the singlet fission dynamics in realistic molecular aggregate systems usually require a larger number of exciton states and vibronic coupling modes. An application of the MCWF approach to SF dynamics of other geometric types of multiradical molecular aggregates including the Peielrs coupling in addition to the Holstein coupling, and extensions to the higher-order unraveling MCWF approach [36] as well as to the non-Markov quantum jump approach [19,37,38] are in progress in our laboratory.
